# Open-source collaboration for *Global Health: Science and Practice*

**DOI:** 10.9745/GHSP-D-13-00012

**Published:** 2013-03-21

**Authors:** Ariel Pablos-Méndez, Michael Klag, Lynn Goldman

**Affiliations:** a Assistant Administrator for Global Health, U.S. Agency for International Development, Washington DC, USA, apablos@usaid.gov; b Dean, Johns Hopkins Bloomberg School of Public Health, Baltimore, MD, USA, mklag@jhsph.edu; c Dean, George Washington University School of Public Health and Health Services, Washington, DC, USA, goldmanl@gwu.edu

## Abstract

USAID and the Schools of Public Health at JHU and GWU welcome you to the inaugural issue of GHSP—an open-access, peer-reviewed journal for the global health community, particularly program implementers, to contribute to and benefit from a dialogue based on science and practical programmatic experience.

It gives us great pleasure to launch this inaugural issue of *Global Health: Science and Practice* (GHSP). Our primary goal is to advance knowledge regarding implementation of global health programs by reaching professionals who design, implement, manage, evaluate, and otherwise support such programs.

This publication represents a collaboration of our institutions—the U.S. Agency for International Development, the Johns Hopkins Bloomberg School of Public Health, and the George Washington University School of Public Health and Health Services. In the spirit of creating a global collaboration, GHSP is completely free and open source—not only for readers but for authors as well. Our Editorial Board reflects the global health community at large. By design, the journal aims to promote easy communication and interactivity. We foresee that public health practitioners, university colleagues, donors, and other development partners will be able to contribute to and benefit from a dialogue based on science and experience of what works for field programs.

GHSP intends to advance implementation science across a wide range of key subject areas with broad remediable impact on health. These will include topics such as maternal and child health, HIV/AIDS, family planning, nutrition, and water and sanitation. We also will tackle the emerging global epidemics of non-communicable diseases and injury. GHSP will take a multidisciplinary approach to the science and practice of global health, bringing together intervention strategies at multiple levels—environmental, population, clinical, and individual—and approaches ranging from strengthening health systems to promoting healthy behaviors.

In the spirit of open communication and scientific integrity, we have embraced the principle of editorial independence as a fundamental tenet of GHSP. We also follow the Uniform Requirements for Manuscripts published by the International Committee of Medical Journal Editors and the code of conduct and best practice guidelines for journal editors from the Committee on Publication Ethics, including scientific peer review.

Global health is at a tipping point. While proud of its accomplishments, the field has embraced bold goals such as ending preventable child and maternal deaths in a generation—among a number of other formidable challenges. We hope GHSP will be a useful and trusted resource instrumental to advancing global health and achieving this ambitious agenda. We look forward to your engagement and feedback.

